# Effects of 3,4-Methylenedioxymethamphetamine on Conditioned Fear Extinction and Retention in a Crossover Study in Healthy Subjects

**DOI:** 10.3389/fphar.2022.906639

**Published:** 2022-07-13

**Authors:** Patrick Vizeli, Isabelle Straumann, Urs Duthaler, Nimmy Varghese, Anne Eckert, Martin P. Paulus, Victoria Risbrough, Matthias E. Liechti

**Affiliations:** ^1^ Clinical Pharmacology and Toxicology, Department of Biomedicine and Department of Clinical Research, University Hospital Basel, Basel, Switzerland; ^2^ Department of Pharmaceutical Sciences, University of Basel, Basel, Switzerland; ^3^ Department of Psychiatry, University of California, San Diego, La Jolla, CA, United States; ^4^ Psychiatric University Hospital, University of Basel, Basel, Switzerland; ^5^ Transfaculty Research Platform Molecular and Cognitive Neuroscience, University of Basel, Basel, Switzerland; ^6^ Laureate Institute for Brain Research, Tulsa, OK, United States; ^7^ Center of Excellence for Stress and Mental Health, San Diego, CA, United States

**Keywords:** MDMA and fear extinction paradigms, fear extinction, skin conductance response, fear-potentiated startle, oxytocin, healthy subjects

## Abstract

**Background:** 3,4-Methylenedioxymethamphetamine (MDMA) has shown initial promise as an adjunct in psychotherapy to treat posttraumatic stress disorder (PTSD). Its efficacy and safety have been demonstrated across phase I–III studies. However, the mechanism underlying the potential utility of MDMA to treat PTSD in humans has not yet been thoroughly investigated. Preliminary evidence suggests that MDMA may facilitate fear extinction recall, which may be through the release of oxytocin. To test this hypothesis, we examined the efficacy of acute MDMA treatment to enhance fear extinction learning and recall.

**Methods:** We used a two-period, double-blind, randomized, placebo-controlled crossover design in 30 healthy male subjects who received a placebo and a single dose of MDMA (125 mg). Fear extinction was tested using two separate Pavlovian fear conditioning paradigms, one using skin conductance response (SCR), and the other fear-potentiated startle (FPS) to conditioned cues. MDMA treatment occurred after fear conditioning and 2 h before extinction learning. Extinction recall was tested 23 h after MDMA intake. Additional outcome measures included subjective effects, emotion recognition tasks, plasma levels of oxytocin, and pharmacokinetics.

**Results:** Fear conditioning and extinction learning were successful in both fear extinction paradigms (generalized eta–squared [ges] for SCR: 0.08; FPS: 0.07). Compared to placebo treatment, MDMA treatment significantly reduced SCRs to the reinforced conditioned stimulus (CS+) during extinction learning (ges = 0.03) and recall (ges = 0.06). Intensity of the subjective effects of MDMA (good effect, trust, and openness) during extinction learning negatively correlated with the discrimination between CS+ and the safety stimulus (CS−) during recall. MDMA did not influence FPS to conditioned cues. Oxytocin concentration was increased fourfold on average by MDMA during acute effects but was not associated with fear extinction outcomes.

**Conclusions:** MDMA treatment facilitated rapid fear extinction and retention of extinction as measured by SCR to fear cues, in line with animal studies of MDMA facilitation of extinction. However, this effect may be limited to certain forms of learned fear responses, as it was not observed in the extinction model using startle reactivity as the outcome. This study provides further evidence for the facilitation of extinction with MDMA treatment and suggests this may be a component of its efficacy when paired with psychotherapy.

**Clinical Trial registration:** clinicaltrials.gov identifier: NCT03527316

## Introduction

3,4-Methylenedioxymethamphetamine (MDMA) is currently being investigated for use as an adjunctive treatment to psychotherapy for patients with PTSD (Oehen et al., 2013; Mithoefer et al., 2019; Jerome et al., 2020; Mitchell et al., 2021) and social anxiety (Danforth et al., 2016). MDMA produces subjective pleasurable effects and alters emotion processing and social cognition in ways that possibly contribute to its potential medical use (Hysek et al., 2012a; Hysek et al., 2014). MDMA-induced inhibition of threat response and facilitation of social responses may be via attenuation of threat circuit (e.g., amygdala) responses to threat stimuli in conjunction with increased reward circuit responses to social stimuli (Bedi et al., 2009). Although the rewarding aspects of MDMA are mediated in part through serotonin release (Liechti et al., 2000; Farre et al., 2007; Hysek et al., 2012b), its effects on oxytocin release may play a role in both its prosocial and threat reduction effects. MDMA stimulates the release of oxytocin (Hysek et al., 2012a; Kirkpatrick et al., 2014), and oxytocin is also known to increase the salience of positive versus negative facial expressions (Marsh et al., 2010), enhance emotional empathy (Hurlemann et al., 2010), and reduced amygdala response to negative emotional stimuli (Kirsch et al., 2005). Oxytocin may be a secondary mediator of MDMA effects on social responses (Vizeli and Liechti, 2018). Furthermore, oxytocin facilitates fear extinction in humans (Acheson et al., 2013; Eckstein et al., 2015) and may therefore contribute to the potential therapeutic effects of MDMA in a patient with PTSD (Mithoefer et al., 2011; Mithoefer et al., 2013; Oehen et al., 2013).

Fear extinction is the process by which a previously learned association between a cue and aversive stimulus is inhibited by a newly learned association. The cue no longer predicts the aversive event. Extinction is thought to be one of the underlying mechanisms of exposure-based therapies, and the first line of treatment for anxiety disorders (Risbrough et al., 2016; Maples-Keller et al., 2022b). Learned fear acquisition and extinction can be tested in animals and humans and is used to probe learned fear processes in anxiety and fear-based disorders such as PTSD (Risbrough et al., 2016). It has been proposed that MDMA facilitation of psychotherapeutic effects for PTSD may be at least in part via the promotion of fear extinction learning (Feduccia and Mithoefer, 2018). Animal studies showed that in mice, MDMA facilitates fear extinction recall (Young et al., 2015), potentially through its serotonin transporter reversal effects and subsequent activation of serotonin 2 A receptors (Young et al., 2017). In contrast, MDMA treatment in rats does not facilitate extinction but instead disrupts fear memory reconsolidation, indicating a more general memory-impairing effect of MDMA (Hake et al., 2019).

Despite the possible approval of MDMA on the drug market, there is only one parallel design study recently published that examined the effects of MDMA on conditioned fear learning and extinction in human subjects (Maples-Keller et al., 2022). However, the effects on extinction were relatively weak, and it was not clear what potential targets of MDMA explain these effects. Thus, the aim of this study was to test the hypothesis that MDMA enhanced extinction recall and that this is through its effects on oxytocin release. We also tested the hypothesis that MDMA plasma levels and associated subjective effects may predict the efficacy of MDMA effects on fear processes, providing potential biomarkers of MDMA target engagement.

## Methods and Materials

### Study Design

The study used a double-blind, placebo-controlled, crossover design with two experimental test sessions to investigate responses to placebo and 125-mg MDMA (corresponding to a mean ± standard deviation [SD] of 1.69 ± 0.17–mg/kg body weight). The order of administration was random and counterbalanced. The washout periods between sessions were >30 days. The study was conducted in accordance with the Declaration of Helsinki and the International Conference on Harmonization Guidelines in Good Clinical Practice and was approved by the Ethics Committee of Northwest Switzerland (EKNZ). The administration of MDMA in healthy subjects was authorized by the Swiss Federal Office for Public Health (BAG), Bern, Switzerland. The study was registered at ClinicalTrials.gov (NCT03527316). The study period was from 30 October 2019 to 23 December 2020.

### Participants

Thirty healthy male participants (mean age ± SD: 26 ± 3.3 years; range: 18–33 years) were recruited through a flyer on the online notice board of the University of Basel or by word of mouth. All the subjects provided written informed consent and were paid for their participation. Exclusion criteria were females (hormonal changes during the menstrual cycle may affect fear acquisition and extinction and would reduce the power of this mechanistic study or increase the number of participants required (Stockhorst and Antov, 2015; Pineles et al., 2016), age ≤18 years or ≥50 years, chronic or acute physical illness (assessed though medical history, physical exam, electrocardiogram, and hematological and chemical blood analyses), personal or family (first-degree relative) history of major psychiatric disorders (assessed by the Semi-structured Clinical Interview for *Diagnostic and Statistical Manual of Mental Disorders*, 4th edition, Axis I disorders), the use of medications that may interfere with the study medications (e.g., any psychiatric medication), tobacco smoking (>10 cigarettes/day), lifetime prevalence of illicit substance use >5 times (except for Δ^9^-tetrahydrocannabinol) or anytime within the previous 2 months and during the study period (determined using urine drug tests). Participants were asked to consume no more than 10 standard alcoholic drinks/week (<120 g ethanol) and have no more than one drink on the day before the test sessions. Subjects had to exhibit a baseline skin conductance (SC) level of 2–20 µS during screening and show a positive response to physiologic stimuli (holding breath) to allow for testing modulatory effects of fear on SC. In addition, when screening for acoustic startle responses, subjects had to show a real-time (30–100 m after the tone) and clear startle response to startling acoustic stimuli (108 dB) in more than 55% (9/16 stimuli). Twenty-nine of 30 participants finished both sessions. One subject did not continue the study after the placebo session and was excluded from the analysis.

### Study Procedures

**FIGURE 1:**
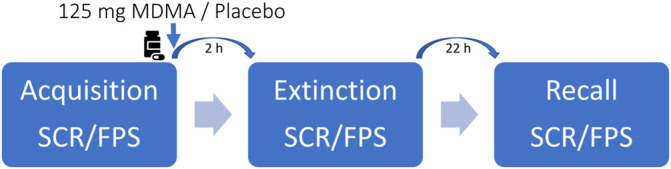
Schematic study day. Participants underwent every two sessions with either 125-mg 3,4-methylenedioxymethamphetamine (MDMA) or placebo. The order was randomized but balanced. The washout period between the study days was at least 30 days. Autonomic and subjective effects were assessed throughout the study day. Blood samples for MDMA and oxytocin blood plasma concentrations were collected before and periodically up to 24 h after drug ingestion.

The study included a screening visit, two 8-h test sessions with a 2-h follow-up session 22 h after drug intake, and an end-of-study visit. Test days were separated by at least 30 days to minimize substance or task-related carryovers. The sessions were conducted in a neutral hospital room. Only one research subject and one investigator were present during each test session. The test sessions began at 8:00 a.m. A urine sample was taken to verify abstinence from drugs of abuse (i.e., opioids, MDMA, amphetamine, cocaine, and tetrahydrocannabinol). The subjects then underwent baseline measurements and fear acquisition training. MDMA or placebo was administered at 10:00 a.m. The outcome measures were repeatedly assessed for 24 h. Fear extinction phases took place 2 h after drug intake. Standardized lunches were served at approximately 1:30 p.m. The subjects were never alone during the acute effect phase. The subjects could go home after the acute effects subsided and return the following day at 8:00 a.m. for the recall phases and follow-up measurements. A schematic study day is displayed in Figure 1.

### Study Drugs

Gelatin capsules that contained 25 mg of pharmaceutically pure MDMA (Lipomed AG, Arlesheim, Switzerland, and ReseaChem, Burgdorf, Switzerland) or a placebo (mannitol) were prepared, randomized, and quality-controlled by a GMP facility (Apotheke Dr. C. Hysek) according to GMP guidelines with authorization from the BAG. The subjects received five capsules with either five times placebo (mannitol) or five times 25-mg MDMA. Participants (mean bodyweight ± SD: 74.5 ± 6.8 kg; range: 55–85 kg) received an individual dose of 1.69 ± 0.17 mg/kg (mean ± SD). Similar dose ranges of MDMA are used in clinical trials (Oehen et al., 2013; Mithoefer et al., 2019).

### Subjective Drug Effects and Effect Durations

To assess subjective alterations in consciousness over time, visual analog scales (VASs) were used repeatedly before and 0, 0.33, 0.66, 1, 1.5, 2, 2.5, 3, 4, 5, 6, and 24 h after drug administration. VASs were presented as 100-mm horizontal lines labeled “not at all” (i.e., 0) on the left side and “extremely” (i.e., 100) on the right side (Hysek et al., 2012b). The following items were used: “any effect,” “good effect,” “bad effect,” “liking,” “high-mood,” “happy,” “fear,” “stimulated,” “content,” “talkative,” “feeling close to others,” “concentration,” “open,” “trust,” and “want to be with other people” or “want to be alone.”

The Adjective Mood Rating Scale (AMRS) (Janke and Debus, 1978) was used before and 1.25, 2, 5, and 24 h after drug administration to assess the mood.

The state-trait anxiety inventory for each state (STAI-S) yields a score for state anxiety levels (Spielberger et al., 1970). The questionnaire was used before, and 1.25, 2, 3, 22.5, and 24 h after drug administration.

### Facial Emotion Recognition Task

The facial emotion recognition task assessed the recognition of basic emotions. The task includes 10 neutral faces and 160 faces that express one of four basic emotions (i.e., happiness, sadness, anger, and fear), with pictures morphed between 0% (i.e., neutral) and 100% in 10% steps. Two female and two male pictures are used for each of the four emotions. Stimuli are shown in random order for 500 ms, followed by the rating screen, where participants have to indicate the correct emotion. The facial images are derived from the Ekman and Friesen series (Ekman and Friesen, 1976).

### Oxytocin Concentrations

Plasma levels of oxytocin were measured at baseline and 2, 3, and 24 h after MDMA administration. Oxytocin concentrations were measured using the oxytocin enzyme-linked immunosorbent assay kit (ENZO Life Sciences, Ann Arbor, MI) according to the manufacturer’s protocol as previously described (Holze et al., 2021). Analyses were performed blinded at the end of the study in one batch.

### Blood Plasma 3,4-Methylenedioxymethamphetamine Concentrations

Blood was collected using lithium heparin tubes. The blood samples were then centrifuged at 4°C at 3,000 rpm for 10 min, and the plasma was stored at −80°C until analysis. MDMA, 3,4-methylenedioxyamphetamine (MDA), and 4-hydroxy-3-methoxymethamphetamine (HMMA) were analyzed in human plasma using high-performance liquid chromatography–tandem mass spectrometry (HPLC–MS/MS). All concentrations were determined after enzymatic deglucuronidation. The lower limit of quantification of MDMA, MDA, and HMMA was 0.5, 1, and 0.5 ng/ml, respectively. A fully validated bioanalytical method was used for the analysis (Dolder et al., 2018).

### Pharmacokinetic Analyses

Pharmacokinetic parameters were estimated using noncompartmental methods. Peak plasma concentration (Cmax) was obtained directly from the observed data. The area under the concentration–time curve (AUC) from 0 to 24 h after dosing (AUC_24_) was calculated using the lin-up, log-down method. The AUC to infinity (AUC_∞_) was determined by extrapolation of the AUC24 using the terminal elimination rate constant (λz). For MDA pharmacokinetics, the λz could not be determined in 17 individuals. Analyses were conducted using Phoenix WinNonlin 8.3 (Certara, Princeton, NJ, United States).

### Autonomic Effects

Blood pressure, heart rate, and tympanic body temperature were repeatedly recorded at baseline and 0, 0.33, 0.66, 1, 1.5, 2, 3, 4, 5, 6, 22.5, and 24 h after drug administration (Hysek et al., 2010).

### Fear Conditioning Paradigms

#### Skin Conductance Response

Fear conditioning using skin conductance response (SCR) was as previously described (Sehlmeyer et al., 2011; Ball et al., 2017). In brief, the unconditioned stimulus (US) was a 1-s long, loud, unpleasant scream (Glenn et al., 2012). The US was delivered by headphones and started 1,500 m after a conditioned stimulus was presented, which then became the paired (fear) conditioned stimulus (CS+). Another conditioned stimulus was never paired with the US and became the neutral (safety) conditioned stimulus (CS−). Between presentations of CS pictures, participants were asked to perform a simple low-level continuous performance task (clicking the right arrow key after appearing of an arrow on the screen). Two sets of each two complex fractal pictures served as either CS+ or CS−. The two fractal pictures per set were counterbalances as CS+ between participants. Stimuli were in color pictures and were presented for 2 s in the center of a black screen. A pseudorandomized order was used such that 1) no more than two successive presentations of the same CS occurred and 2) the CSs were equally distributed within each half of the acquisition period. The paradigm consisted of four phases: a brief familiarization phase, the acquisition phase, the extinction phase, and the extinction recall phase. The familiarization phase involved five presentations of each CS with no instances of the US. The acquisition phase was conducted completely before dosing and broken into two runs of 8-min each. Each run consisted of 15 presentations of the CS− and 20 presentations of the CS+: 5 (25%) with (CS+ paired) and 15 without (CS + unpaired) the US. The extinction phase was assessed 2 h after drug administration, falling during peak plasma levels of MDMA. Extinction recall was assessed the next day, 23 h after drug administration. The extinction learning and recall phases involved 25 and 15 presentations of each CS with no instances of the US, respectively. Participants were not informed about the aim of the test. SCRs were recorded with two electrodermal activity (EDA) finger electrodes (TSD203) via the skin conductance unit (EDA100C-MRI) and the acquisition module MP160 (Biopac Systems Inc. Goleta, CA, United States). The electrodes were fixed with gel mimicking the salt concentration of sweat (GEL101) and were placed on the middle and index fingers of the nondominant hand. The data were recorded and analyzed with AcqKnowlege 5.0.1 software (Biopac Systems Inc. Goleta, CA, United States). SCR amplitudes were determined as the maximum response 0.5–6 s after the CS onset. The SCR amplitudes were measured from baseline to peak. The threshold level for an SCR to be considered a valid response was 0.01 µS. No valid response to a CS was imputed as 0 µS. Final data processing consisted of z-transformation per subject, session, and phase to mitigate interindividual variability and normalize the distribution. Outliers of ±2 standard deviations were excluded and imputed with a moving average (i.e., 5.4% of the data) (Eckstein et al., 2015). Five participants with no valid responses in the placebo session to either CS+ or CS− in at least one phase (i.e., acquisition, extinction, or recall) were considered nonresponders and excluded from the within-subject analysis. One subject had no valid response in both sessions and was therefore excluded from all SCR analyses.

#### Fear-Potentiated Startle

The FPS paradigm has been described before in detail (Acheson et al., 2015; Straus et al., 2017). The fear conditioning and extinction task is comprised of three phases. The acquisition phase was conducted before dosing, and the extinction learning phase was conducted 2.5 h after dosing. The extinction recall was performed the next day, 23.5 h after drug administration. The eye-blink startle response was measured using electromyography (EMG) startle system (EMG100C) and the acquisition module MP160 (BIOPAC Systems, Inc. Goleta, CA, United States). Signal detection was adjusted to a 100–1,000 Hz range. All electrode resistances were <10 kΩ. Participants sat upright in a chair in a quiet testing room for assessments of startle reactivity. To measure startle reactivity, two small cup electrodes (Ag/AgCl) were placed below and lateral to the left eye over the orbicularis oculi muscle. During testing, two colored symbols were presented to the participants on a computer screen. One symbol (CS+) was paired with an aversive stimulus, and the other symbol was presented without the US (CS−). A 500-ms, 250-psi air puff to the larynx served as the aversive stimulus. During the acquisition session, the CS+ was presented with the air puff 75% of the time. During the extinction and recall phase, the air puff was never presented. To test the fear acquisition and extinction of the CS +, participants heard short white-noise tones (108 dB) that elicited a startle response during the CS+, CS− presentations, and between the CS presentations as baseline startle measurement (NA trials). The colored symbols were counterbalances as CS+ between participants. Following each phase, self-reported anxiety was assessed by asking how anxious participants felt in the presence of the CS+ and CS− (1–10 scale). Startle data was initially processed by averaging responses to CS+, CS−, and NA trials within each phase into blocks of two trials each (Straus et al., 2017). This resulted in startle scores for each stimulus across four blocks of acquisition, eight blocks of extinction, and four blocks of recall. To standardize startle values and reduce between-subjects variability, the average of NA trials per phase was subtracted from each CS+, CS−, and NA block and then divided by the SD of NA trials within each phase (resulting in a z-score normalized to the baseline in each phase for each subject). Those scores were Winsorized at 5 (values greater or smaller than ±5 were trimmed to 5/−5, i.e., 2.7% of the data). At last, NA blocks were subtracted from their respective CS+ and CS− blocks to obtain values representing standardized potentiated startles above baseline for each CS type within each block (Richards et al., 2022). To assess contingency awareness, participants were asked to report at each stimulus whether they expected to receive an air puff now (+1), they were unsure (0), or they did not expect an air puff (−1).

### Statistical Data Analysis

Subjective and autonomous maximum (Emax) and minimum (Emin) effects were determined directly from the observed data. Area und the effect-time curve from 0 to 24 h (AUEC_24_) after drug administration was calculated using the trapezoidal method. Repeated measure analyses of variance (rmANOVA) were performed for the FPS and SCR data, and significant main effects and interactions were followed by Tukey posthoc comparisons. RmANOVAs were performed in the computer software JASP (JASP Team, 2022). Drug condition, time, and stimulus type served as within-subject factors. Pearson correlations (coefficient, R_P_) and paired *t*-tests (*t* value, t_p_) were performed in the statistical analysis software R (R Core Team, 2021). Maulchy’s test for sphericity indicated no violation of the assumption of sphericity. The SCR and FPS data is shown as z-scores per subject, drug condition, and phase. Data shown in the correlation matrixes are during MDMA sessions only and not corrected for multiple testing. The level of significance was set at *p* < 0.05. Generalized eta square (ges) values show effect sizes.

## Results

### Fear Induced Skin Conductance Response vs. Fear-Potentiated Startle

**FIGURE 2:**
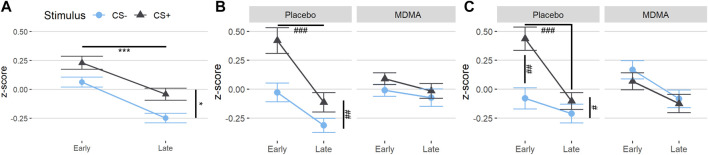
MDMA treatment reduces CS+/CS− discrimination during fear extinction learning and enhances extinction recall. Standardized values (z-scores) of both trial types (conditioned fear stimulus [CS+] and conditioned safety stimulus [CS−]) of the skin conductance response task. **(A)** Subjects showed differentiation between CS+ and CS− in acquisition. **(B)** In the extinction learning phase, subjects after a placebo displayed discrimination of the conditioned stimuli in the early phase and extinction over time, but already early nondiscrimination of the conditioned stimuli after MDMA. **(C)** In the recall phase, subjects after the placebo displayed an early return of fear memory by discrimination of the conditioned stimuli. There was no discrimination of the conditioned stimuli after MDMA, indicating extinction retention. The acquisition was before drug intake. MDMA, 3,4-methylenedioxymethamphetamine. Values are mean ± standard error of the mean (SEM). Stats in **(A)** are main effects, *p < 0.05, ***p < 0.001. Stats in **(B)** and **(C)** are from Tukey posthoc tests, ^#^ *p* < 0.05, ^##^ *p* < 0.01, ^###^ *p* < 0.001.

**FIGURE 3:**
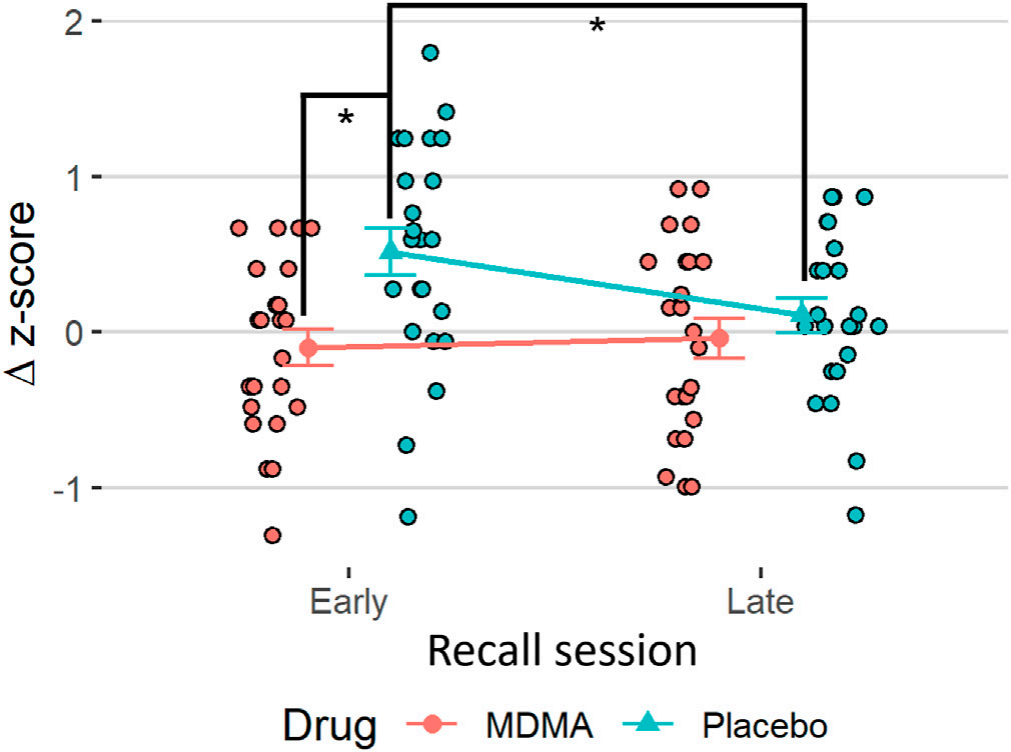
MDMA promotes extinction recall. The difference in standardized values (Δz-scores) between the trial types (conditioned fear stimulus [CS+] minus conditioned safety stimulus [CS−]) of the skin conductance response task in the recall. Subjects after MDMA showed significantly less discrimination between CS+ and CS− compared to the placebo session. Stats are from Tukey posthoc tests following an interaction of drug × time in delta values [(F1,22) = 6.74, *p* = 0.017]. *p < 0.05. MDMA, 3,4-methylenedioxymethamphetamine.

During acquisition, SCR responses were greater to CS+ than to CS−, indicating successful conditioning, although there was an overall diminution of responses to both cues over the session likely reflecting some habituation (Figure 2A, main effect of time: F (1,22) = 41.1, *p* < 0.001, ges = 0.17, and main effect of trial type: F (1,22) = 5.76, *p* = 0.025, ges = 0.08). The same time effect was observed for the extinction learning and recall phase, with a decrease in magnitude from early to late recall phase [F (1,22) = 17.6, *p* < 0.001, ges = 0.11 and F (1,22) = 16.7, *p* < 0.001, ges = 0.12, respectively]. During the extinction learning (Figure 2B), a main effect for trial type was observed [F (1,22) = 8.42, *p* = 0.008, ges = 0.08] as well as an interaction of drug × trial type F (1,22) = 5.18, *p* = 0.033, ges = 0.03, and drug x time [F (1,22) = 4.68, *p* = 0.042, ges = 0.05)]. Tukey posthoc comparison showed that SCR across trial types only differed in the placebo condition (*p* = 0.004), but not under the influence of MDMA (*p* = 0.80). Independent of trial type, SCRs during early extinction learning were higher than in late extinction, but only in the placebo session (*p* < 0.001) and not in the MDMA session (*p* = 0.81). Baseline SC levels during acute MDMA treatment were higher than placebo (Supplementary Figure S1, mean ± SD: 10.1 ± 3.6 vs. 6.6 ± 2.3 µS, t_p_ = 4.83, *p* < 0.001, ges = 0.26). In the recall phase, however, the response to trial type only tend to differ (*p* = 0.089), but there was an interaction between drug and trial (F (1,22) = 5.26, *p* = 0.032, ges = 0.06), as well as with drug × trial × time [F (1,22) = 6.74, *p* = 0.017, ges = 0.02, Figure 2C]. Tukey posthoc comparison showed that SCR responses to CS+ and CS− only significantly differed in the placebo condition (*p* = 0.029), but not after MDMA treatment (*p* = 0.92). During the first half of the recall phase, SCR responses in the placebo session were higher to CS+ than CS−, (*p* = 0.003) and compared to SCR during CS+ in the last half of extinction recall (*p* < 0.001). The difference between CS+ and CS− during the extinction recall phase per drug session is displayed in Figure 3. MDMA treatment was associated with significantly smaller difference between SCR to CS+ vs CS− in the early recall phase compared to placebo (*p* = 0.013). Only participants after the placebo session had fear memory recovery, which extinguished overtime (*p* = 0.024, meaning no recall of extinction), while participants after the MDMA session showed no difference in responses to CS+ and CS− trials (*p* = 0.92). All the results were similar in the nonstandardized data but with greater variability (Supplementary Figure S2). The participants were engaged in the task as evidenced by 99 ± 1% (mean ± SD) accuracy in the simple low-level continuous performance task in both drug conditions.

**FIGURE 4:**
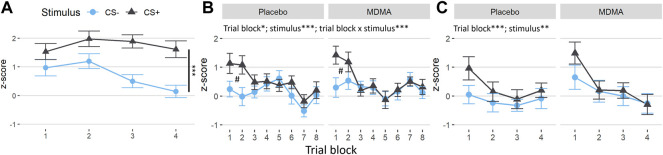
No changes induced by MDMA in the fear-potentiated startle task. Difference between the standardized values of both conditioned trial types (conditioned fear stimulus [CS+] and conditioned safety stimulus [CS−]) and the pulse alone (NA) during the fear-potentiated startle task. **(A)** Subjects showed a clear differentiation between CS+ and CS− in acquisition. **(B)** In the extinction learning phase, subjects in both sessions displayed discrimination of the conditioned stimuli in the early trial blocks (^#^CS+ vs. CS− of block 1 and 2, Tukey posthoc: *p* < 0.001 and *p* < 0.01, respectively) and extinction over time (trial blocks). **(C)** In the recall extinction phase, subjects in both sessions showed a return of fear of the conditioned stimulus in the early trial blocks and renewed extinction over time. The acquisition was before drug intake. MDMA, 3,4-methylenedioxymethamphetamine. Values are mean ± SEM. Stats in **(A)** are main effects. *p < 0.05, **p < 0.01, ***p < 0.001.

FPS showed a significant main effect of trial type during acquisition, [Figure 4A; F (1,28) = 36.5, *p* < 0.001, ges = 0.07], as well as during extinction learning [Figure 4B; F (1,28) = 14.7, *p* < 0.001, ges = 0.01] and extinction recall [Figure 4C; F (1,28) = 11.5, *p* = 0.002, ges = 0.01], with FPS to the CS+ being higher relative to the CS−, indicating successful fear conditioning. There was a main effect for time in the extinction learning [F (7,196) = 2.7, *p* = 0.010, ges = 0.03] and extinction recall [F (3,84) = 6.0, *p* < 0.001, ges = 0.03], but not in the acquisition phase. During extinction learning, there was a time × trial type interaction, with conditioned fear responses to the CS+ decreasing significantly across the extinction phase (Figure 4B, time × trial type interaction: F (7,196) = 4.17, *p* < 0.001, ges = 0.02. FPS was significantly higher in the first two CS+ trial blocks compared to CS− trials (both *p* < 0.004), and compared to CS+ during later blocks (*p* = 0.037–<0.001). Startle response across trial types, test phases and overall was not affected by MDMA treatment (neither standardized nor nonstandardized, Supplementary Figure S3). Participants’ reported expectancy of the US (air puff) also declined across extinction blocks, and this effect was not different across drug condition [Supplementary Figure S4; time main effect: F (1,28) = 39.4, *p* < 0.001, ges = 0.09].

### Autonomic and Subjective Effects

**FIGURE 5:**
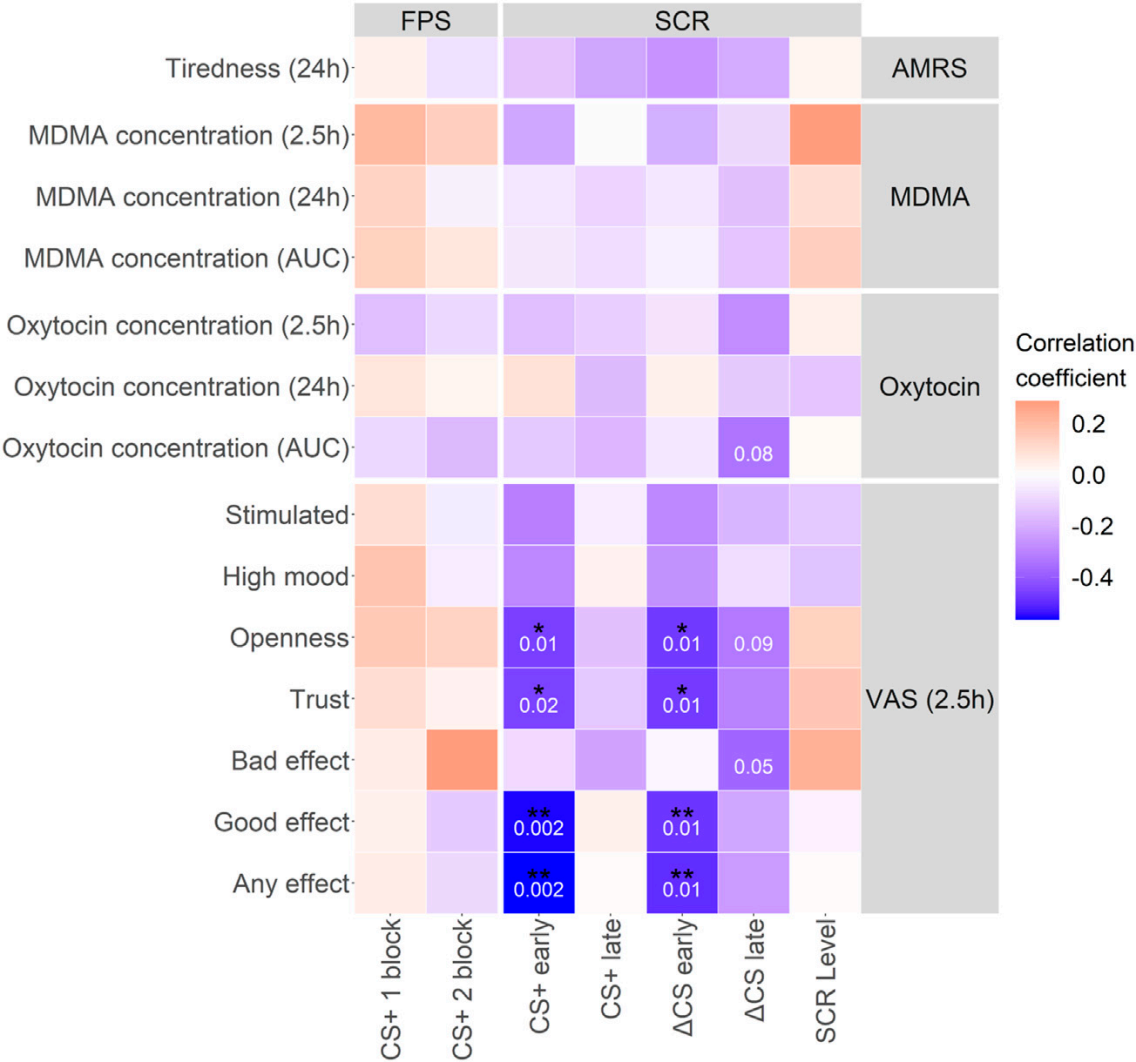
MDMA characteristic effects, such as trust and openness, correlate with extinction recall. Correlation matrix between MDMA and oxytocin concentrations and subjective effects of MDMA with extinction recall parameters. Characteristic MDMA effects during extinction learning correlated with the CS+ and subsequently ΔCS in the early phase of extinction recall. Pearson correlation coefficient was used. *p*-values in white numbers. *p < 0.05; **p < 0.01. Not corrected for multiple testing. CS+, conditioned fear stimulus; CS−, conditioned safety stimulus; ΔCS, CS+–CS−; AMRS, Adjective Mood Rating Scale; VAS, visual analog scale; FPS, fear-potentiated startle; SCR, skin conductance response; MDMA, 3,4-methylenedioxymethamphetamine. Concentrations at 2.5 h were generated as the mean of the respective 2 and 3 h time points. Data is from MDMA sessions only.

MDMA moderately increased all measured vital parameters (i.e., blood pressure, heart rate, and body temperature) as shown in Supplementary Figure S5A,B and Supplementary Table S1. MDMA-induced higher ratings in subjective effects, such as any drug effect, “good” drug effect, liking, and talkative, as shown in Supplementary Figure S5C and Supplementary Table S1. MDMA produced significantly higher maximum scores (mean ± SD: 39 ± 5.3 vs. 36 ± 5.1; t_p_ = 3.15, *p* = 0.004, ges = 0.09) and also lower minimum scores (mean ± SD: 28 ± 4.1 vs. 30 ± 4.3; t_p_ = 2.33, *p* = 0.027, ges = 0.05) on the real-time anxiety index STAI compared with placebo. The SC level response under the acute influence of MDMA was significantly higher than under placebo (Supplementary Figure S1) and was significantly associated with MDMA plasma concentrations (Supplementary Figure S6; R_P_ = 0.47, *p* = 0.010). The day after MDMA treatment there were no differences in SC level across placebo and MDMA treatment. Subjective effects, such as any drug and good drug effect, as well as openness and trust during the extinction learning phase negatively correlated with the CS+ and ΔCS z-score in early extinction recall (Figure 5: any drug: R_P_ = −0.57, *p* = 0.002 and R_P_ = −0.5, *p* = 0.007, good drug: R_P_ = −0.56, *p* = 0.002 and R_P_ = −0.48, *p* = 0.010, openness: R_P_ = −0.46, *p* = 0.015 and R_P_ = −0.47, *p* = 0.012, trust: R_P_ = −0.45, *p* = 0.016 and R_P_ = −0.47, *p* = 0.012, respectively).

### Pharmacokinetics

Pharmacokinetic results are displayed in Supplementary Table S2 and Supplementary Figure S7.

### Oxytocin

MDMA produced significantly higher blood plasma oxytocin levels compared to placebo (Supplementary Figure S8A; mean ± SD; Cmax: 502 ± 271 vs. 125 ± 100 pg/ml, t_p_ = 6.70, *p* < 0.001, ges = 0.49; AUC_0–24h_: 6,742 ± 3,235 vs. 2,489 ± 1,537 pg*h/ml, t_p_ = 6.70, *p* < 0.001, ges = 0.48). The total amount and the amount of MDMA at 2 h in the blood plasma positively correlated with the total amount and the amount of oxytocin at 2 h (Supplementary Figures S8A, S9B); R_P_ = 0.41, *p* = 0.025, and R_P_ = 0.42, *p* = 0.024, respectively). The oxytocin concentration at the time of the emotion task (3 h) did not correlate with the emotion recognition (Supplementary Figure S9A) or with the fear extinction learning results (Supplementary Figure S6).

### Facial Emotion Recognition Task

**FIGURE 6:**
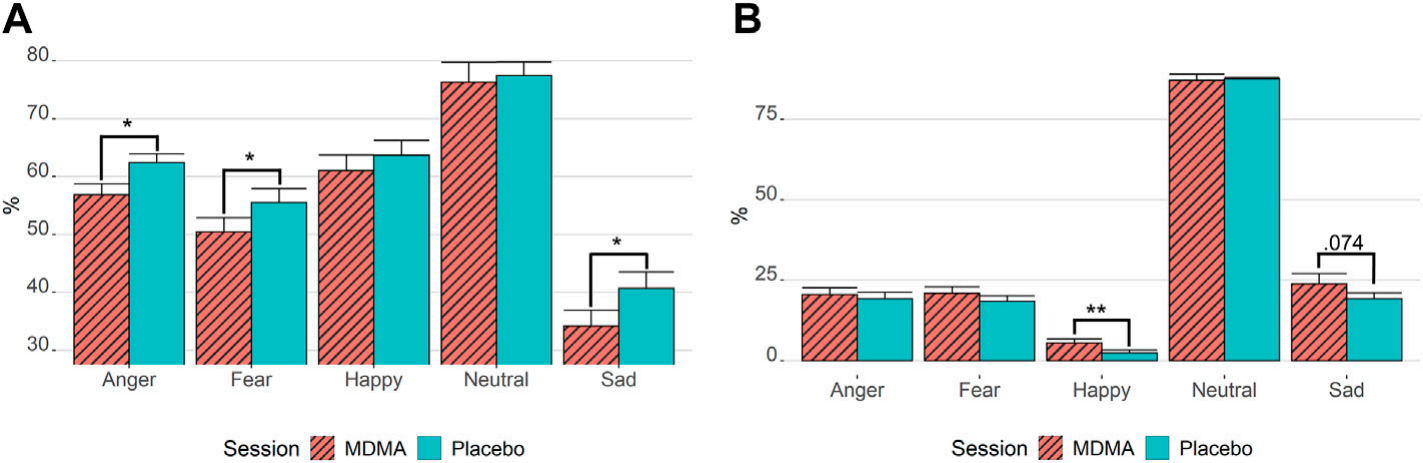
MDMA inhibits the recognition of negative emotion and promotes misclassification as happy. Results of the facial emotion recognition task. **(A)** Correctly identified emotions. **(B)** Emotion misclassification if not correctly recognized. Stats are from paired *t*-tests. *p < 0.05; **p < 0.01. Bonferroni corrected for multiple testing. MDMA, 3,4-methylenedioxymethamphetamine. Values are mean ± standard error of the mean. The task was performed 3 h after drug intake.

As displayed in Figure 6A, rmANOVA showed a drug effect for the correct identification of emotions, with participants under the influence of MDMA performing worse than participants in the placebo condition [F (1,28) = 12.3, *p* = 0.002, ges = 0.02]. The identification performance of each emotion under MDMA influence vs placebo revealed that MDMA significantly impaired the recognition of faces displaying negatively valanced emotions, such as “anger” (t_p_ = 3.21, *p* = 0.015, ges = 0.09), “sad” (t_p_ = 3.02, *p* = 0.025, ges = 0.05), and “fear” (t_p_ = 2.85, *p* = 0.040, ges = 0.04), but not “happy” or “neutral.” Most emotions that were not correctly identified were misclassified as “neutral” (Figure 6B). The fewest trials were misclassified as “happy.” However, when analyzing the misclassification as “happy” for drug session differences, participants under the influence of MDMA misinterpreted faces significantly more as “happy” (t_p_ = 3.77, *p* = 0.004, ges = 0.07). The dependent *t*-test *p*-values were Bonferroni corrected for all (5) emotions.

### Order and Sequence Effects

There was no order or sequence effect observed in subjective or autonomic effects. In the SCR and EMG FPS measurements, an order effect was discovered in the nonstandardized data [F (1,28) = 9.85, *p* = 0.005 and F (1,28) = 6.81, *p* = 0.014, respectively], with overall lower values in the second session. Self-reported expectancy of the US was also generally higher in the first than the second session [F (1,28) = 16.4, *p* < 0.001]. However, there was no drug × order or trial type × order interaction, neither in the standardized nor in the nonstandardized data (all *p* > 0.52), indicating that there was no carryover effect.

## Discussion

The present study provides the first within-subject comparison of fear extinction learning and recall between MDMA and placebo. There were three main effects. First, MDMA treatment increased extinction recall as measured using SCR. Acute subjective effects of MDMA were significantly associated with increased extinction recall in this paradigm, suggesting subjective effects may be a rapid and feasible marker of MDMA effects on extinction targets. Second, contrary to our hypothesis, we did not observe an association between peripheral oxytocin levels and extinction recall, suggesting that oxytocin release, at least in the periphery, is not linked to MDMA effects on extinction. Third, the effects of MDMA on extinction were limited to the SCR fear conditioning paradigm but not on enhanced recall of extinction in the FPS paradigm.

The main finding was that participants showed a stronger fear extinction recall in the SCR paradigm the day after MDMA treatment, compared to participants after placebo. Acquisition of conditioned fear was successful, as indicated by the main effect of trial type in both tasks (SCR and FPS). The acquisition recall and subsequent extinction of the conditioned fear were also successful, as indicated by the early discrimination of trial type and the later absence of discrimination during extinction learning. Only in the early extinction phase of the SCR task, participants did not discriminate between CS+ and CS−. On the one hand, this could indicate rapid extinction learning or a failing of fear memory recall. In contrast to studies with MDMA in mice and oxytocin in humans suggesting immediate enhancement of fear extinction learning and recall (Eckstein et al., 2015; Young et al., 2015; Young et al., 2017), in rats, it was observed that fear memory reconsolidation may be disrupted when injecting MDMA after fear memory reactivation (Hake et al., 2019). However, there is no data on MDMA injection shortly after fear memory acquisition. Given the administration of MDMA immediately after acquisition training, one could argue MDMA could have a general consolidation disrupting effect. On the other hand, SC levels were elevated under the acute influence of MDMA. This is most likely a direct representation of sympathomimetic activation using MDMA since SC reflects the change in sweat gland activity (Hysek et al., 2013; Brown and Pollard, 2021). Oftentimes, MDMA concentration in blood plasma correlated with SC levels. A similar potentially misleading stimulant-related effect was observed in rodents: rats did not freeze after high MDMA injections during extinction learning (Hake et al., 2019). Therefore, the nondiscrimination of stimuli during acute MDMA effects should be interpreted with caution, as potential ceiling effects cannot be excluded.

Nevertheless, the same nondiscrimination of trial type was observed during extinction recall 24 h after MDMA treatment and when baseline SC was no longer different between drug and placebo treatment. In addition, the intensity of the MDMA effects on self-reports on feelings of trust and openness during the fear extinction learning correlated with the fear extinction retention. Assessments of the intensity of the subjective effects of MDMA have previously been associated with a decrease in blood flow in the right amygdala and hippocampus (Carhart-Harris et al., 2015). Thus, it is possible that the subjective effects of MDMA could be a predictor of treatment efficacy for extinction or other threat-circuit–related behaviors in PTSD (Shin et al., 2006). However, one could argue that both the subjective effects and the BOLD signals are orthogonal and simply related to MDMA pharmacokinetics.

Similar to a recently published randomized controlled parallel study by Maples-Keller et al. (2022b). we did not find significant changes in FPS after MDMA treatment. However, Maples did note that MDMA treatment increased the prevalence of extinction “retainers” vs. “nonretainers” at extinction recall, using an arbitrary cutoff method to identify these groups. Using the same cutoffs, we were unable to detect a difference in the prevalence of retainers vs. nonretainers in the current study. We did not see an increase in extinction retainers in the current study because we used a stronger and longer US (air puff), which may have made the fear association more resistant to drug effects on extinction (500 ms at 250 psi vs. 250 ms at 140 psi). Another difference between this and the study by Maples-Keller et al. (2022b) is the timing of the acquisition and recall phase. While they conducted acquisition 24 h before and recall training 48 h after extinction learning, we chose a period of 2 h before and 24 h after, respectively. Nevertheless, the results of the two studies are overall comparable. Both studies demonstrated the feasibility of experimental extinction research under the influence of MDMA (100 and 125 mg). Participants were able to navigate through and were engaged in the task, as evidenced by 99% accuracy in the simple, low-threshold, continuous performance task during conditions. Furthermore, both studies found no clear facilitation of fear extinction learning per se but rather a consolidation of extinction learning at extinction recall, albeit in different tests. Other similar findings of this study and that of Maples-Keller et al. (2022b) are that MDMA transiently causes an increase in cardiovascular activity and a slight increase in body temperature compared with placebo. Consistent with our previously reported data, the drug was overall well-tolerated and predominantly positively perceived (Vizeli and Liechti, 2017).

Although there is a growing body of evidence that the benefits of MDMA for anxiety disorders are based on learning to extinguish learned fear, another important aspect is the entactogenic and empathogenic effects of MDMA (Feduccia and Mithoefer, 2018). MDMA generates dose-dependent, positive drug effects such as “good drug effect,” “openness,” “high-mood,” and “trust” (Schmid et al., 2014; Holze et al., 2020; Studerus et al., 2021). Entactogenic effects, such as trust and openness, might promote and strengthen the bond between therapist and patient (Mithoefer et al., 2011). These effects may also make it easier for the patient to access anxious memories without yielding to overwhelming emotions, thus improving therapeutic success in psychotherapy. These considerations underline the importance of subjective effects not only as a possible predictor of fear extinction retention but also as a possible catalysator of therapist–patient interaction. A recent study has suggested that exposure to antidepressant drugs could decrease the treatment responsiveness to MDMA in psychotherapy (Feduccia et al., 2020). Although these results were not confirmed in a larger analysis (Mitchell et al., 2021), they would be consistent with previous findings of selective serotonin reuptake inhibitors (SSRIs; e.g., citalopram) blocking MDMA-induced subjective effects in humans (Liechti and Vollenweider, 2000) and fear extinction enhancement in mice (Young et al., 2017).

The empathogenic effects of MDMA are thought to increase the ability to recognize positive feelings in others (Hysek et al., 2012a). In line with previous studies, we showed that participants under the influence of MDMA were generally worse at correctly identifying negative facial expressions (i.e., “anger,” “fear,” and “sad”) and more often misinterpreted them as happy faces (Schmid et al., 2014; Bershad et al., 2019). The release of oxytocin is thought to be at least partially responsible for these characteristic effects of MDMA (Dumont et al., 2009). However, despite our observation of a fourfold MDMA-induced increase in oxytocin concentration, similar to previous studies (Holze et al., 2020), we found no correlation with facial emotion recognition. In addition, we did not find a correlation between peripheral oxytocin levels and extinction. This suggests that MDMA does not, or at least not exclusively, rely on oxytocin to facilitate fear extinction. We demonstrated, however, a correlation between some subjective effects (e.g., openness) and blood plasma oxytocin concentration after 2 h. In contrast, many studies have not found a relationship between oxytocin concentration and the subjective, emotional, empathic, or prosocial effects of MDMA (Hysek et al., 2012a; Hysek et al., 2014; Kirkpatrick et al., 2014; Kuypers et al., 2014; Parrott, 2016). This may be in part because the reliability of oxytocin measurements can vary substantially, and sample sizes should be high (>100) to achieve acceptable power (Martins et al., 2020). Furthermore, plasma oxytocin levels may also not reflect oxytocin levels in the brain (Neumann et al., 2013). To further investigate the mediating role of oxytocin in the subjective, empathogenic, or fear-inducing effects of MDMA, the use of an oxytocin receptor antagonist that can cross the blood–brain barrier, such as retosiban, may be required. The study has certain limitations. First, we only observed drug effects in the SCR task, whereas the FPS task did not show any drug-related changes. While SCR is an excellent way to measure arousal, it is less specific for emotional valence (i.e., fear) (Lang et al., 1998). PTSD symptoms were also found to be more strongly associated with deficits in the extinction using FPS than SCR (Glover et al., 2011; Norrholm et al., 2011). This differential effect across the two protocols may be due to relatively high extinction recall in the FPS paradigm in the placebo group (e.g., ceiling effects) or differences in MDMA effects across measures of fear responding. However, in addition, sympathomimetic activation led to increased sweating during the acute effect in the extinction learning phase. To avoid interference with SCR, future studies could investigate the feasibility of other compounds with a less stimulating profile than MDMA but similar therapeutically beneficial properties (e.g., R-MDMA instead of racemic) in fear extinction (Curry et al., 2018; Oeri, 2020). Second, a crossover design is especially useful to eliminate interindividual differences for the primary outcome, but it also has its drawbacks, especially in tasks involving learning. There was a clear order effect with less reaction in the second session. However, due to counterbalancing the sessions and a washout period of at least 30 days, this was limited to order, and no carryover effects were observed. Third, the possibility of self-unblinding in a study with a psychoactive substance is a general concern. However, the use of an active placebo, while appropriate in some studies, could affect the mechanism of fear learning or extinction itself (Brignell and Curran, 2006). Fourth, sex differences were not addressed in this study. The study design did not include females due to concerns that hormonal influences on fear extinction might reduce the power to detect drug effects on extinction (Stockhorst and Antov, 2015; Pineles et al., 2016). More recent studies indicate, however, that there are no sex differences in response to MDMA effects on extinction nor in response to MDMA effects on PTSD symptoms (Mithoefer et al., 2019; Mitchell et al., 2021; Maples-Keller et al., 2022b). Future research will be important to investigate MDMA and the impact of menstrual hormones in fear extinction learning, especially since women are disproportionally more at risk of developing an anxiety disorder than men (Breslau et al., 1997). In addition, emotion recognition may vary between the sexes. While there is limited evidence of a discrepancy in recognition of basic emotions (except for disgust; Connolly et al., 2019), the administration of MDMA showed a stronger impairment of negative emotion recognition in women than in men (Hysek et al., 2014). However, the exogenous administration of oxytocin showed conflicting trends in men (Domes et al., 2007) vs. women (Domes et al., 2010). At last, we included only healthy and young volunteers without psychiatric disorders. The true potential of MDMA may lie particularly in its efficacy in people with fear extinction deficits, such as patients with PTSD, which warrants further investigation within this population.

## Conclusion

MDMA displayed nondiscrimination of opposing conditioned stimuli in the extinction training and extinction recall in the SCR model, indicating beneficial effects in fear extinction learning and retention. This effect correlated with the intensity of the MDMA effects during extinction learning. However, we did not observe the same fear extinction facilitating effect in the FPS test. This study provides further compelling information about the mechanism of action that may be involved in the beneficial effect of MDMA in psychotherapy. Further mechanistic research in patients is needed to determine the full range of MDMA’s supportive effects.

## Data Availability

The datasets presented in this article are not readily available because the data associated with this work are owned by the University Hospital Basel and were licensed by Mind Medicine. Requests to access the datasets should be directed to ML, matthias.liechti@usb.ch.
