# Supplementary material for: Effects of 3,4-Methylenedioxymethamphetamine on Conditioned Fear Extinction and Retention in a Crossover Study in Healthy Subjects
Source: Front Pharmacol. 2022 Jul 13;13:906639. doi: 10.3389/fphar.2022.906639 (PMC9326355; doi:10.3389/fphar.2022.906639)
Supplement: Supplementary file 1 [file DataSheet1.docx]

**Supplementary Material**

**Effects of MDMA on conditioned fear extinction and retention in a cross-over study in healthy subjects**


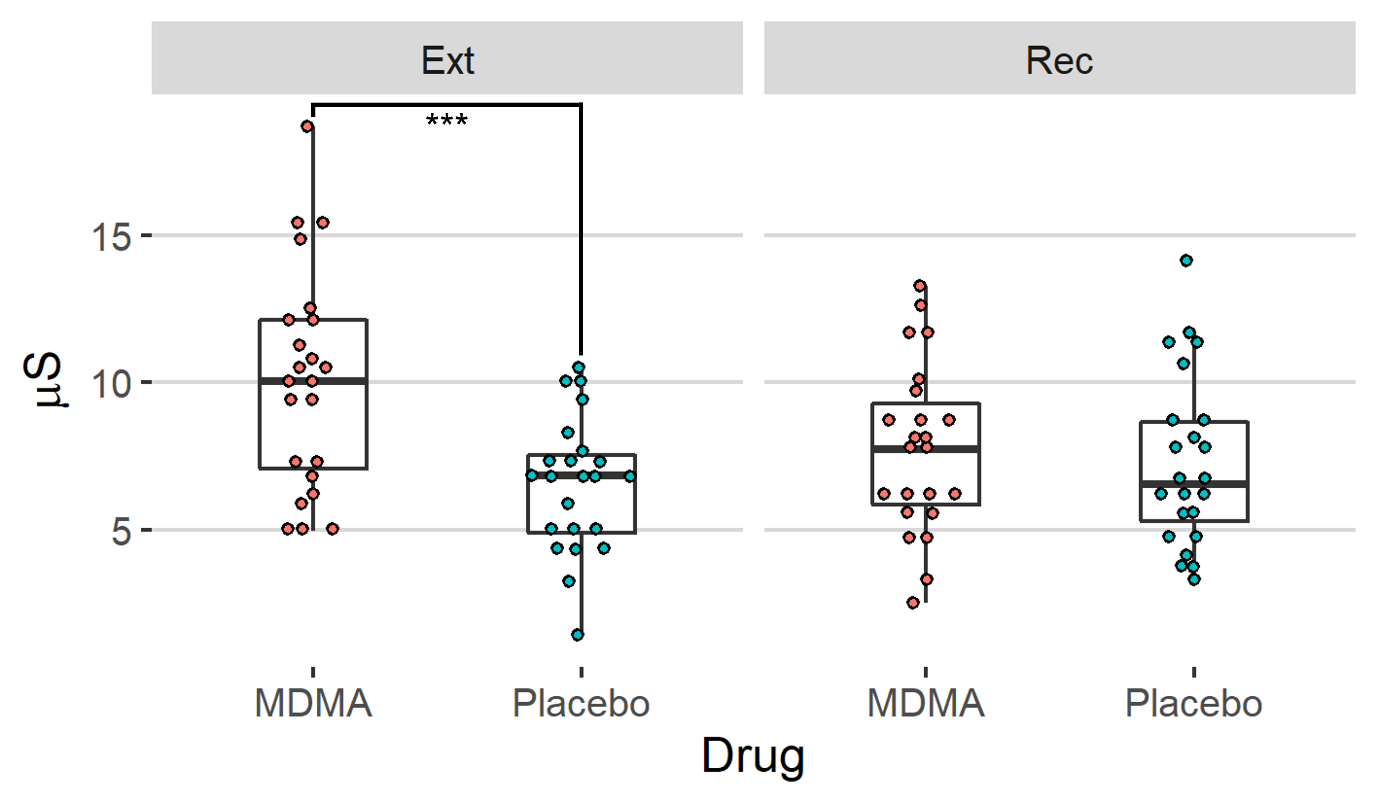


**Supplementary Figure S1** Average skin conductance baseline level in extinction training (Ext) and extinction recall (Rec). Stats are from paired t-test. *** p < .001.


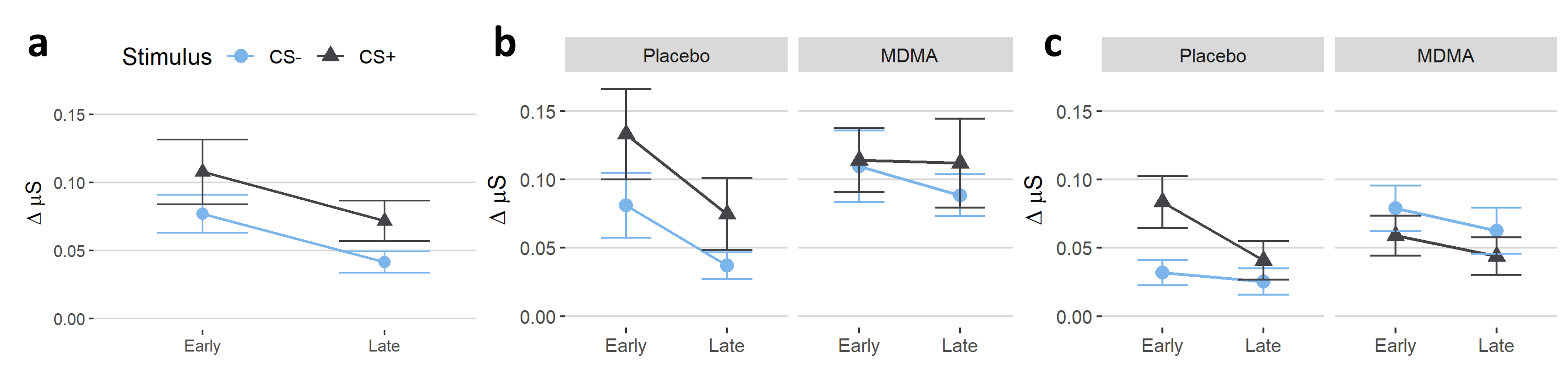


**Supplementary Figure S2** Non-standardized values (baseline to peak in micro Siemens) of both trial types (conditioned fear stimulus [CS+] and conditioned safety stimulus [CS-]) of the skin conductance response task in **(a)** Acquisition, **(b)** Extinction, **(c)** Recall. Acquisition was before drug intake.


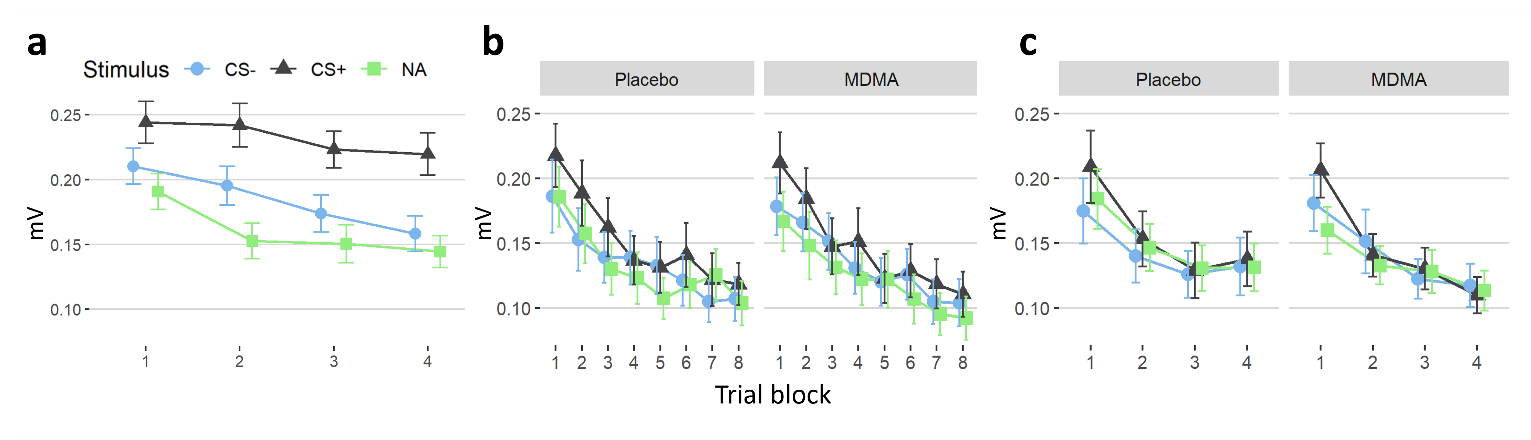
**Supplementary Figure S3** Non-standardized values (millivolts) of all trial types (pulse alone [NA], conditioned fear stimulus [CS+], and conditioned safety stimulus [CS-]) of the fear-potentiated startle task in **(a)** Acquisition, **(b)** Extinction, **(c)** Recall. Acquisition was before drug intake.


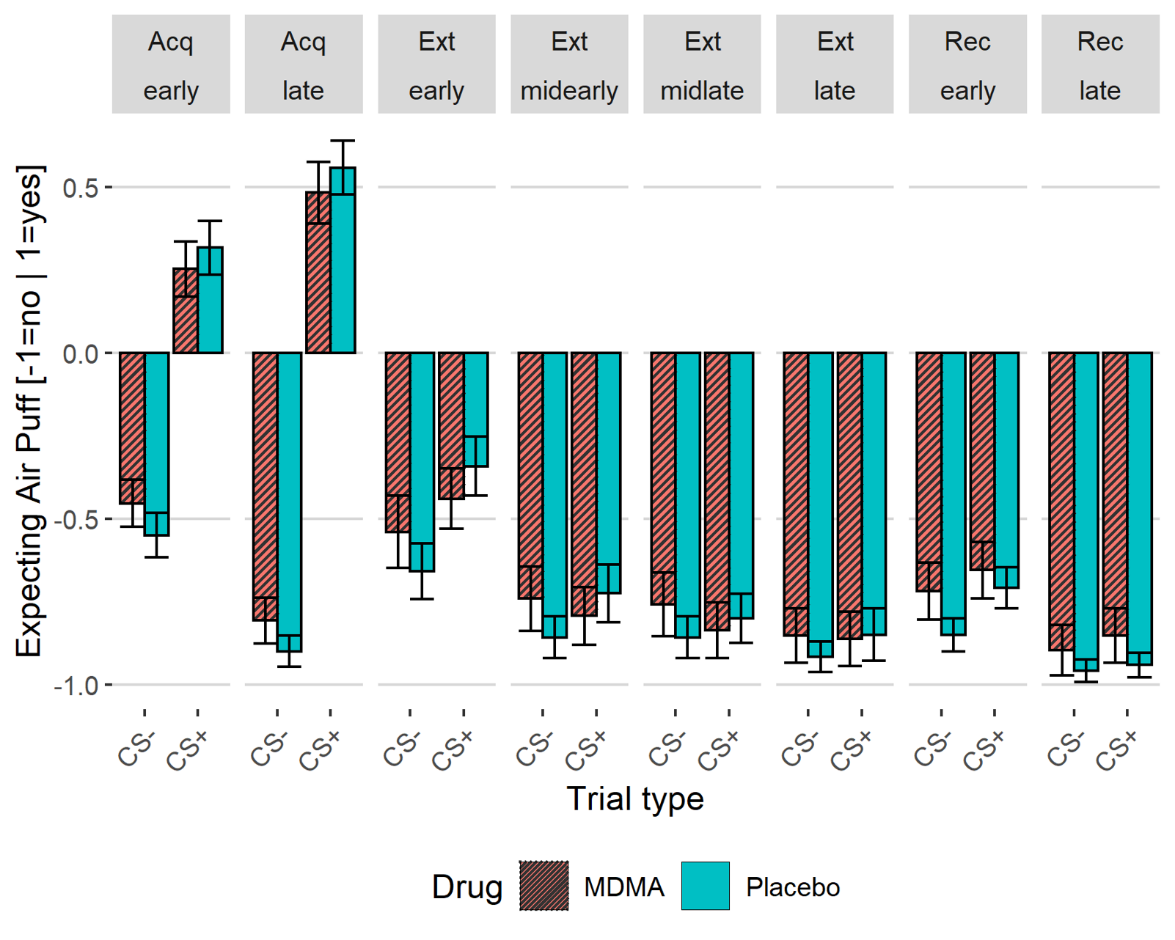


**Supplementary Figure S4** Unconditioned stimulus (US) expectancy task during fear-potentiated startle test. Participants were asked to report at each trial whether they expected to receive an US (i.e. air puff) now (+1), they were unsure (0), or they did not expect the US (-1). Acq, acquisition; Ext, extinction; Rec, recall.


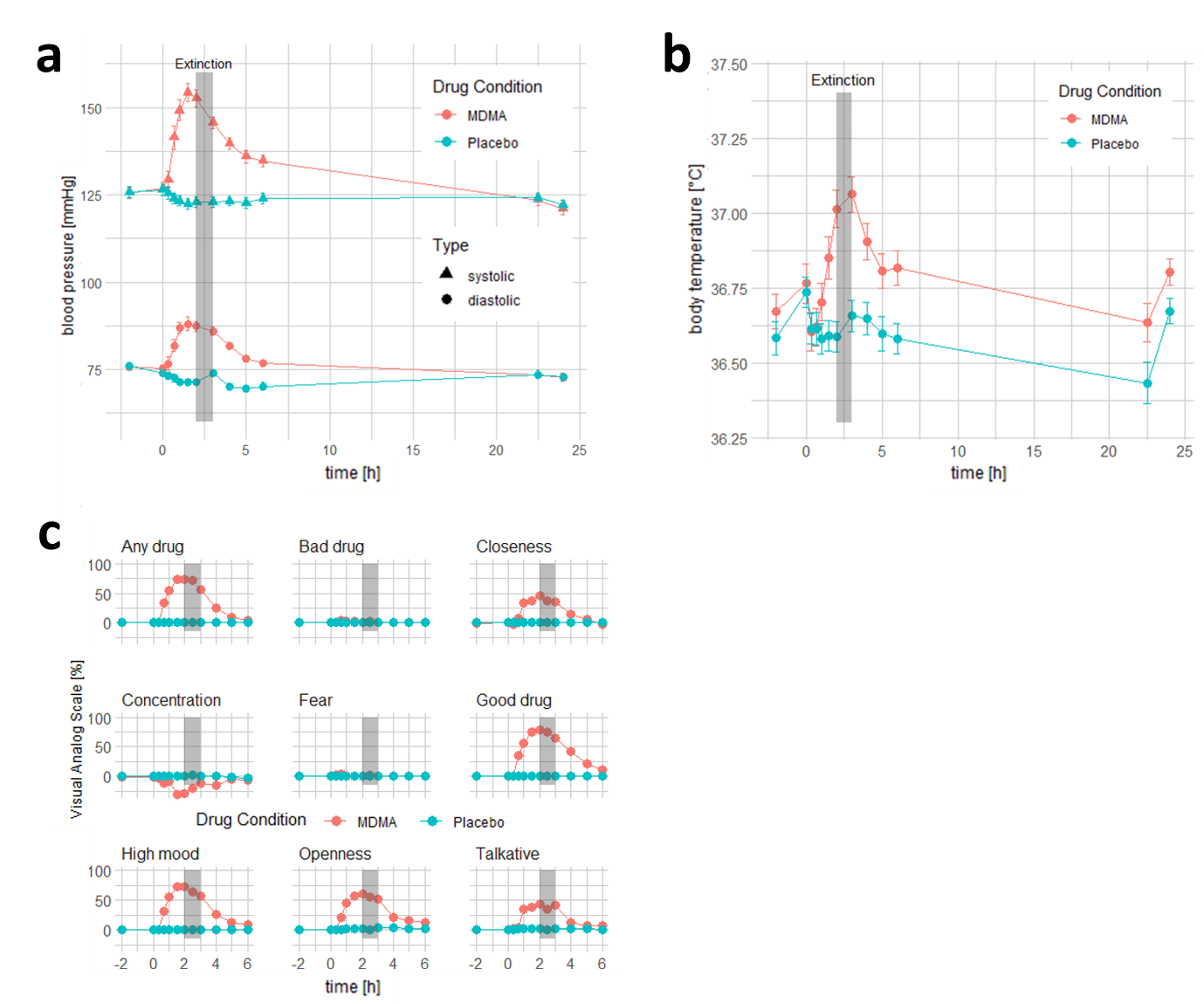


**Supplementary Figure S5** Autonomous and subjective effects of 125 mg MDMA / Placebo given at time 0 h. **(a)** systolic and diastolic blood pressure. **(b)** body temperature. **(c)** subjective effects captured by Visual Analog Scales. Extinction learning phase was between 2 – 3 h after drug intake. Stats in Supplementary Table 1.


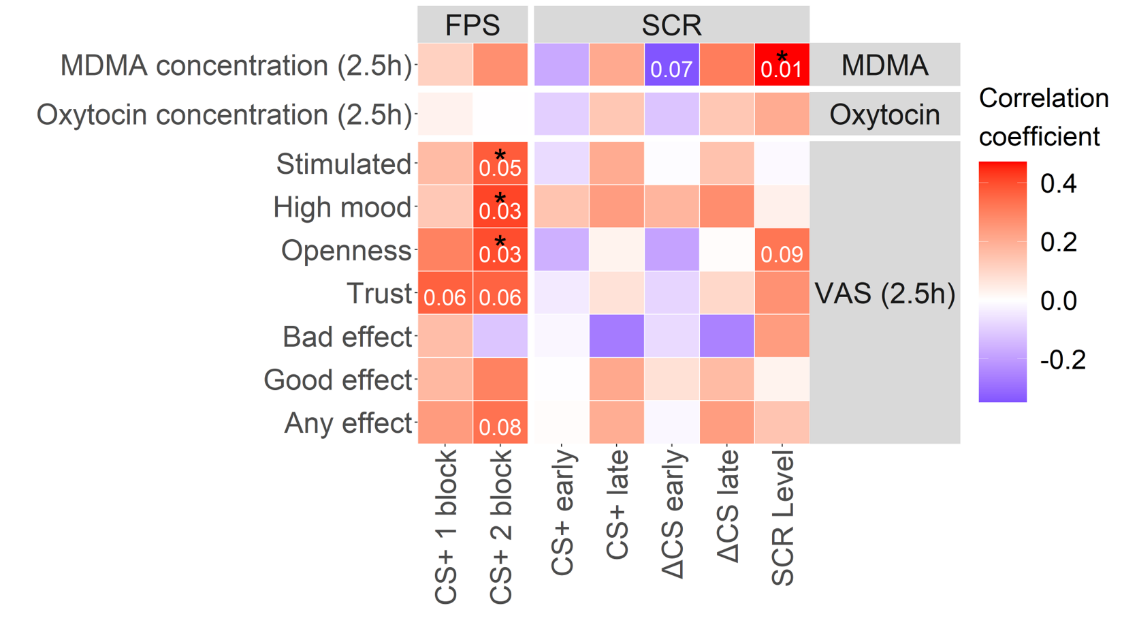


**Supplementary Figure S6** Correlation matrix between MDMA and oxytocin concentration, and subjective effects during extinction learning and extinction learning parameters. Pearson correlation coefficient was used. P values in white numbers. * p < .05. The displayed results are exploratory and p-values are not corrected for multiple testing. ΔCS, CS+ - CS-; VAS, visual analog scale; FPS, fear-potentiated startle; SCR, skin conductance response. Concentrations at 2.5 h were generated as mean of the respective 2 and 3 h time point. Data is from MDMA sessions only.


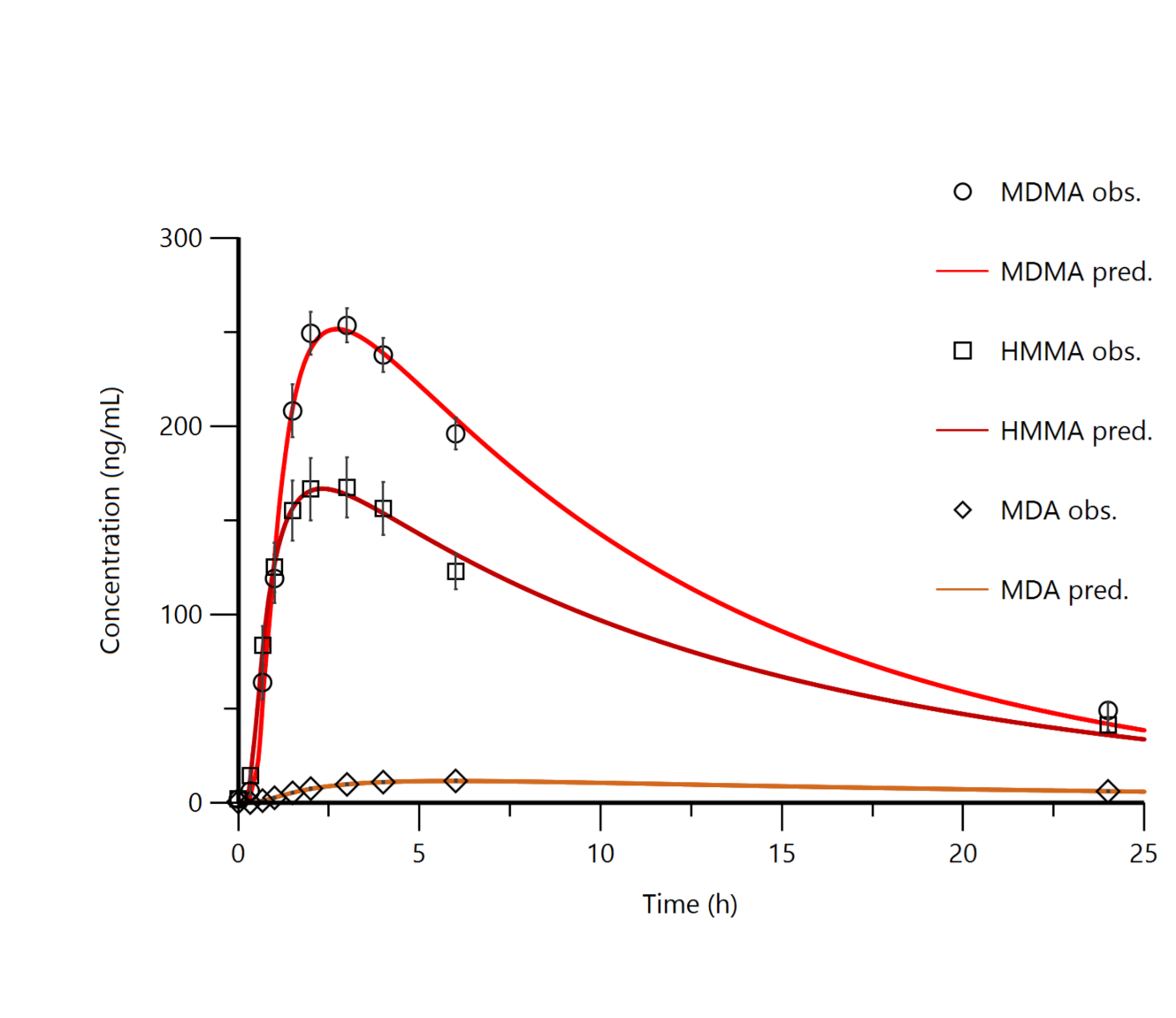


**Supplementary Figure S7** Blood plasma concentration of MDMA and its main metabolites HMMA and MDA. A one-compartment model was used with first-order input and first-order elimination. Initial estimates were given by the prior non-compartmental analyses. The model fit was assessed by visual inspection and Akaike information criteria. The HMMA model fit was impaired without lag time and not relevantly improved by a two-compartment model. Obs., observed data; pred., predicted data. MDMA, 3,4-Methylenedioxymethamphetamine; HMMA, 4-Hydroxy-3-methoxymethamphetamine; MDA, 3,4-Methylenedioxyamphetamine


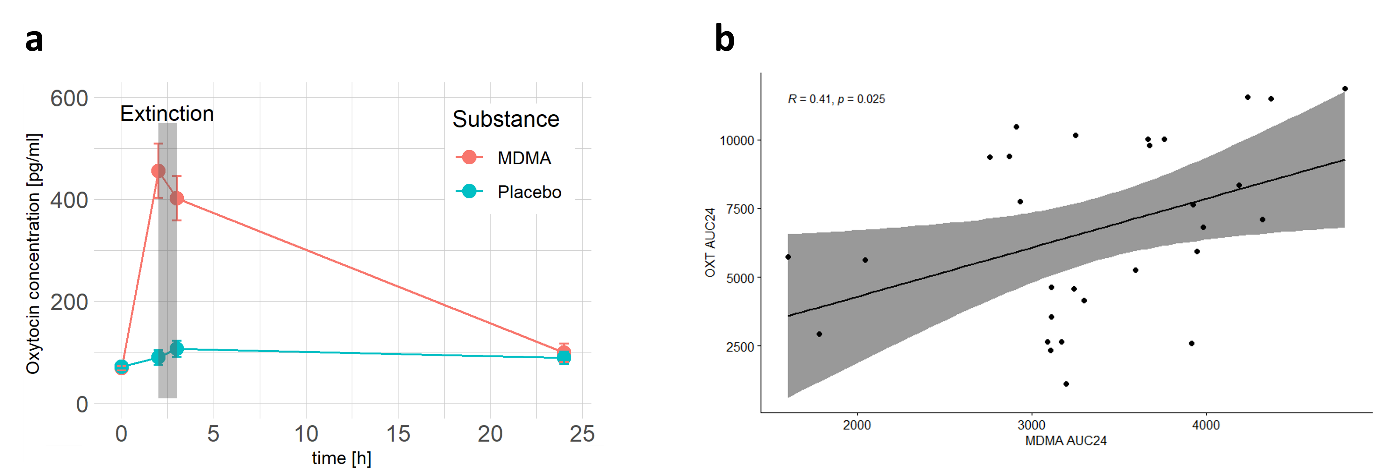


**Supplementary Figure S8 (a)** Oxytocin blood plasma concentration after 125 mg MDMA / Placebo given at time 0 h. **(b)** Pearson correlation of the area under the concentration-time curve (AUC) from 0 – 24 h of oxytocin and MDMA. Extinction learning phase was between 2 – 3 h after drug intake.


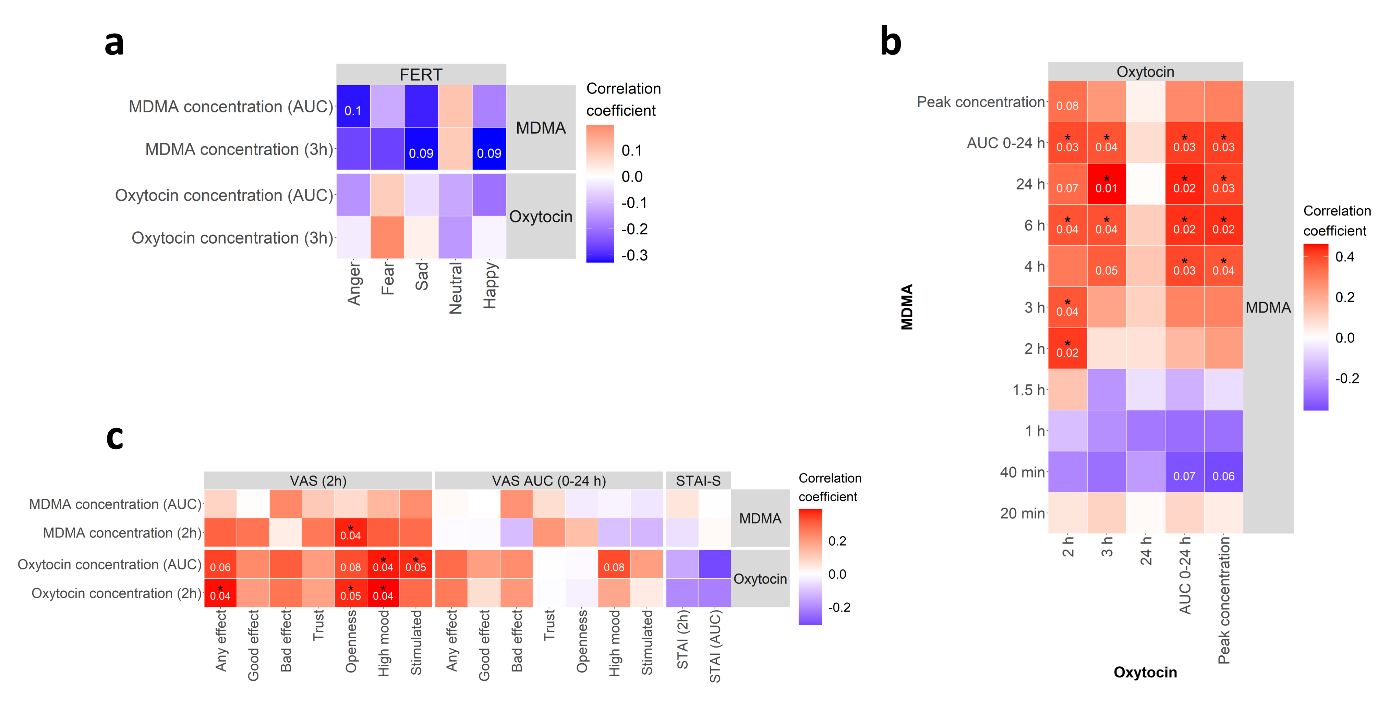


**Supplementary Figure S9** Correlation matrix of: **(a)** MDMA and oxytocin concentration with facial emotion recognition task (FERT) correct answers. **(b)** MDMA with oxytocin blood plasma concentrations at different time points and overall. **(c)** MDMA and oxytocin concentration with subjective effects. Pearson correlation coefficient was used. P values in white numbers. * p < .05. The displayed results are exploratory and p-values are not corrected for multiple testing.. ΔCS, CS+ - CS-; VAS, visual analog scale; FPS, fear-potentiated startle; SCR, skin conductance response; AUC, area under the concentration-time curve; STAI-S, state-trait anxiety inventory for state. Concentrations at 2.5 h were generated as mean of the respective 2 and 3 h time point. Data is from MDMA sessions only.

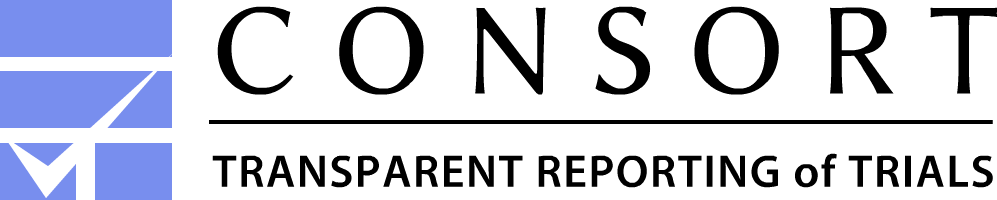


Assessed for eligibility (n=35)

**CONSORT 2010 Flow Diagram**

Allocated to MDMA (n=15)

♦ Received allocated intervention (n=15)

♦ Did not receive allocated intervention (n=0)

## Follow-up

Lost to follow-up (n=0)

Discontinued intervention (n=0)

Lost to follow-up (n=0)

Discontinued intervention (n=1)

1. Personal issue

Allocated to placebo (n=15)

♦ Received allocated intervention (n=15)

♦ Did not receive allocated intervention (n=0)

## Allocation

## Period 1

## Enrollment

Randomized (n=30)

Excluded (n= 5)

♦  Not meeting inclusion criteria (n= 3)

♦  Declined to participate (n= 2)

♦  Other reasons (n= 0)

## Allocation

## Period 2

Allocated to MDMA (n=14)

♦ Received allocated intervention (n=14)

♦ Did not receive allocated intervention (n=0)

Allocated to placebo (n=15)

♦ Received allocated intervention (n=15)

♦ Did not receive allocated intervention (n=0)

## Follow-up

Lost to follow-up (n=0)

Discontinued intervention (n=0)

Lost to follow-up (n=0)

Discontinued intervention (n=0)

Analysed (n=29)
♦ Excluded from analysis (n=0)

## Analysis
